# Supplementary material for: Prevalence of myopia among disadvantaged Australian schoolchildren: A 5-year cross-sectional study
Source: PLoS One. 2020 Aug 27;15(8):e0238122. doi: 10.1371/journal.pone.0238122 (PMC7451552; doi:10.1371/journal.pone.0238122)
Supplement: Attachment — Submitted filename: PONE-D-20-09893 Response to Reviewer Comments.docx [file pone.0238122.s002.docx]

Dear Dr Wang

Thank you for the opportunity to revise this manuscript for consideration. The comments have been carefully considered and a revised submission has been uploaded. Changes to the manuscript are highlighted in yellow and detailed responses to the reviewers’ comments are presented in italics below.

Reviewer #1:

It's a known fact, nothing new. Study of myopia, astigmatism separately, not using SER may be more important in the future. Full cycloplegic refraction data will be needed. Different age groups will be also needed to be separated.

*Answer: (1) Thank you for this comment. We have separated the astigmatic component as suggested and presented this by age (in Tables 1 and 2).*

*We have added this information to lines 187-201, page 8: “The overall mean astigmatism was -0.16 ± 0.39D (range 0 to -5.00D). Seventy-nine percent of eyes exhibited negligible astigmatism (i.e. ≤0.25 D) and 18% showed astigmatism between -0.25 D and -1.0 D. Multivariate regression analyses showed that the mean astigmatism was not related to sex (β=0.002, 95% CI: -0.024~ 0.028, P =0.89), age (β= -0.01, 95% CI: -0.02~ 0, P = 0.27) and year of testing (all P > 0.05). Of the 893 (21%) eyes with astigmatism >0.25 D in the current study, approximately 53% exhibited with-the-rule astigmatism (WTR: negative axis within 30° of the horizontal), nearly 5% exhibited oblique astigmatism (axis 30 - 60° or 120 - 150°) and the remaining 42% exhibited against-the-rule astigmatism (ATR: axis 60 - 120°). Across the three time points, the prevalence of WTR increased from 37.9% to 60.6% and 64.4% in 2014, 2016 and 2018, respectively (P < 0.001). In comparison, over the 3 years, ATR prevalence decreased from 55.8% to 34.9% and 31.0%, respectively (P < 0.001), oblique astigmatism prevalence was 5.3%, 6.4% and 4.6%, respectively. (P = 0.51).”*

*(2) This was a screening study and cycloplegic refraction was not determined. This limitation has been addressed in the discussion at lines 308-319, page12: “Cycloplegic refraction is proposed as the gold standard for determining refractive error and yields better results than non-cycloplegic retinoscopy. Non-cycloplegic retinoscopy can result in overestimation of myopia and underestimation of hyperopia in young children. However, if we remove the 6-7 age group or 6-8 age group from the analysis, the overall trend of myopia prevalence with age does not change, indicating that this overestimation is unlikely to have occurred. In this study, the screening eye examination was part of a battery of other physical examinations, consequently cycloplegic refraction was not appropriate. In several previous Australian studies, non-cycloplegic refraction was performed in a similar age groups in a similar manner allowing a meaningful comparison over time.”*

Reviewer #2:

This is a detailed study, but as you've mentioned, this study didn't use cycloplegic refraction. Since the overall spherical equivalent refraction was less than 1 diopter, the amount of refraction changed might be significant.

*Answer: Please see the response to question 2, reviewer #1.*

Reviewer #3:

1. In this study, refractive error was determined by noncycloplegic retinoscopy, instead of cycloplegic autorefractometry. Noncycloplegic refraction could result in overestimation of myopia and underestimation of hyperopia, especially in young children. Therefore, there would be some impacts in the statistical results. For example, in table 2, the higher prevalence of myopia in age group 6-7 than that in age group 8 could be the result of pseudomyopia in younger kids. In table 1, higher proportions of younger children (age 6-8) in 2014 group might result in more overestimation of myopia.

*Answer: (1)* *Noncycloplegic refraction may indeed result in overestimation of myopia and underestimation of hyperopia, especially in young children. We have further analysed the data based on age. In Table 2, there are 4(2.5%) and 5(1.7%) children with myopia in age 6-7 group and in age 8 group respectively. If we remove the 6-7 age group* or 6*-8 age group, the overall trend of myopia prevalence with age does not change.*

*A sentence has been added at lines 312-314 as follows:* “However, if we remove the 6-7 age group or 6-8 age group from the analysis, the overall trend of myopia prevalence with age does not change, indicating that this overestimation is unlikely to have occurred.”

*In Table 1, there are 2(1%), 3(2%) and 4(3.6%) children with myopia aged 6-8 in 2014, 2016 and 2018, respectively. Although there are more children aged 6-8 in 2014, the proportion of myopia is not statistically higher. Meanwhile, we found that the mean SER decreased slightly and the mean* *astigmatism was stable from 2014 to 2018.*

2. Children with ocular pathology were excluded from the study, but strabismus cases were not excluded. Without cycloplegia, the real refractive status could be masked by accommodation in strabismic children (ex. accommodative esotropia, intermittent exotropia ...).

*Answer: Thank you for this comment. We excluded the 112 strabismus cases (64 exotropia, 47 esotropia, 1 vertical strabismus) and reanalyzed the data. We added this content and changed all other relevant data throughout the manuscript. See lines 119-122, page 4: “the data of 4365 (95.2%) children were used for analysis. Of the 220 children whose data were excluded (67, 67 and 86 cases in 2014, 2016 and 2018, respectively), 112 were strabismus (64 exotropia, 47 esotropia, 1 vertical strabismus)”. Meanwhile, the conclusions of the article did not change after excluding the112 strabismus cases compared with including them.*

3. The author did not mention that the retinoscopy was performed by one or multiple examiners. There would be inter-observer variability of retinoscopy.

*Answer: Thank you for this comment. All the retinoscopy was performed by one examiner (AD). We added this content on lines 93-94, page 4.*

4. The author did not mention the composition of subjects. As we know, race is an important factor of myopia prevalence.

*Answer: Thank you for this comment. Please see the links (*<https://en.wikipedia.org/wiki/Asian_Australians>*) for specific data on Asians lived in Australia.  The table "Metropolitan areas with significant Asian Australian populations (2016 Census)” seems to indicate that 97.7% of Asians live in the capital cities.* *Our participants are from rural and outer suburban areas and Asian children are a minority. This has been addressed in the discussion at lines 261-264(Our participants are from rural and outer suburban areas and Asian children are a minority and the ethnic mix of participants has not changed significantly over the period of the study).*

5. There is no discussion about the significantly increased proportions of both myopia and hyperopia in this study as compared to the similar study in 1976.

*Answer: The prevalence of myopia in Australia is increasing, consistent with reports from many other parts of the world. The main reason of the increased proportions of myopia may be the changing educational system and lifestyle. The slightly higher proportion of hyperopia compared to the earlier studies is consistent with another recent study in the Sydney urban population and is discussed at lines 272-279.*

Reviewer #4:

The authors of the paper “Prevalence of myopia among disadvantaged Australian schoolchildren: a 5-year cross-sectional study “presents a cross-sectional analysis of refractive data and myopia prevalence in Australian children during the years 2014/2016/2018. The authors found that, although the prevalent of myopia remained low comparing with other locations, the mean spherical equivalent refraction (SER) decreased from 2014 to 2018, accompany with shifting towards less hyperopia. They also found that females had higher mean SER, but with faster decrease than male with age. In general, this paper is well-written and easy to follow. However, there are few points to be considered.

1. This paper found the mean SER decreased from 2016 to 2018, with trends of less hyperopia and stable myopia. However, reviewing the numbers of included cases, there are less children enrolled in the low grade groups in 2018, with more children enrolled in the middle to high grade groups, comparing with 2014 and 2016 (ex. Only 1.9% in age group 6 and 7 in 2018 vs. 4.7% and 4% in 2014 and 2016, respectively). Could the authors make some explanations that this will not lead to selection bias?

*Answer: Thank you for this comment. The School Principal selects all eligible children from disadvantaged families, so there is some variation in the age and sex profile of children year to year. However, multivariate regression analyses accommodate these variations in numbers by adjusting for age and sex to estimate the changing trend of refractive error over time (Table 3). We have added this information to lines 319-327, page 12: “ In addition, there are less children enrolled in the low-grade groups in 2018, with more children enrolled in the middle to high grade groups, comparing with 2014 and 2016 (ex. Only 1.9% in age group 6 and 7 in 2018 vs. 4.7% and 4.1% in 2014 and 2016, respectively). However, the School Principal selects all eligible children from disadvantaged families, so there is some variation in the age and sex profile of children year to year. Meanwhile, multivariate regression analyses accommodate these variations in numbers by adjusting for age and sex to estimate the changing trend of refractive error over time.”*

2.The authors found females had higher mean SER, but with faster decrease with age comparing with male. Unlike other studies with higher prevalence of myopia in female, the higher prevalence of hyperopia in female in the present study is somewhat different. This could possibly be explained by different visual experiences in daily life. However, similar to question 1, there are more female children included in this study, especially in 2014 and 2016, could this also be a confounding factor?

*Answer: Thank you for this comment. As for the question regarding age, multivariate regression analyses accommodate these variations by adjusting for age and sex to estimate the changing trend of refractive error over time (Table 3).*
