# Supplementary material for: Prevalence of myopia among disadvantaged Australian schoolchildren: A 5-year cross-sectional study
Source: PLoS One. 2020 Aug 27;15(8):e0238122. doi: 10.1371/journal.pone.0238122 (PMC7451552; doi:10.1371/journal.pone.0238122)
Supplement: Attachment — Submitted filename: PONE-D-20-09893 Response to Reviewer Comments.docx [file pone.0238122.s003.docx]

“*Prevalence of myopia among disadvantaged Australian schoolchildren: a 5-year cross-sectional study”*

*We thank the reviewers again for their helpful suggestions and offer this rejoinder in response to the comments.*

Reviewer #2:

(1) I wonder that why did you choose to remove the 6-7 age group or 6-8 age group from the analysis for supporting your opinion?

*Response:*

*During the first round of comments on the manuscript, the third reviewer raised a question as follows ‘*Noncycloplegic refraction potentially resulting in overestimation of myopia and underestimation of hyperopia, especially in young children. Therefore, there would be some impacts in the statistical results. For example, in table 2, the higher prevalence of myopia in age group 6-7 than that in age group 8 could be the result of pseudomyopia in younger kids. In table 1, higher proportions of younger children (age 6-8) in 2014 group might result in more overestimation of myopia.” T*o address this question, we reanalyzed the data with either the 6-7 age group or 6-8 age group removed. The overall trend of myopia prevalence with age did not change. This has been described at line 315 and we have made no further changes to the manuscript in this revision.*

(2) To my knowledge, previous study suggested that cycloplegia refraction should be performed before age 20, because the differences might up to 0.36±0.41D in the 13 year olds group (IOVS April 2010).

*Response:*

*In the study referred to (IOVS April 2010),^1^ refractive error before and after cycloplegia was measured using a Humphrey 598 Autorefractor. They compared the difference between refractive error before and after cycloplegia resulting in the conclusion above. However, a different method was used in our study: refractive error was determined by non-cycloplegic retinoscopy using the fogging technique while the child maintained fixation on a distant (6 m) non-accommodative target.* *Yeotikar ^2^ found that mean difference in SER for 7 to 16 years children obtained by non-cycloplegic refraction with contralateral fogging and cycloplegic refraction was small (mean of 0.29D) and was not clinically significant. We have added this content to the section on limitations in the discussion (line 312-315).*

*We agree that with respect to physiological aspects, the absence of cycloplegia (thus allowing accommodation as an uncontrolled variable) does affect one’s power to understand the degree of refractive latency to a certain degree. However, the dogmatic use of cycloplegics in all studies as dictated by the RESC protocol is not warranted, particularly in a screening examination in a respite environment as described in this study. The percentage of children affected by this latency is quite small and will have a very small impact on the mean SER of a cohort of thousands of children.*

*Furthermore, the mandatory inclusion of cycloplegia avoids the issue arising from a number of reports alluding to the fact that cycloplegia can physiologically cause a significant minus shift in refractive error in a (albeit small) percentage of children (e.g. FA Young etal 1971, WM Ludlam et al 1972, HC Cheng & YT Hsieh 2014) and adults (TT Toh et al 2005). Other reports do not comment on this aspect, yet their data clearly indicate the same message when visually inspecting their scatter plots showing the difference in SER pre/post cycloplegic (e.g. R Fotedar etal 2007, where all dots below the x-axis represent children exhibiting a minus shift during cycloplegia). This minus-wards shift is due presumably to a forward shift of the crystalline lens during cycloplegia (L Gao etal 2002). Notably, the percentage of children shifting from one RESC-designated refractive group to another as a result of cycloplegia is very small, either plus-wards or minus-wards, indicating that net relative shift in numbers due to the physiological variable of accommodation does not necessarily mean that the any overall claims driven by the data will change substantially.*

*As other non-cycloplegic studies also exist in the literature, we believe there are sufficient data for direct comparison with the results presented in this study and have not made further changes in the manuscript.*

Reviewer #3: The analysis in this study is detailed. However, there are two weaknesses.

1. Noncycloplegic refractive examination was used.

*Response:*

*See above for Response to Reviewer 1, Q (2)*

2. There are a lot of similar studies in the literature, including quite a few studies of Australian children. This study did not provide anything new.

*Response：*

*The other Australian data either represents a complete cross section of society (Sydney Myopia study data),^3-5^ or, comes mainly from higher socioeconomic groups (VEC).^6,7^ Our new data focusses more on lower socioeconomic groups, and the lower prevalence of myopia in those children from predominantly rural regions corresponds with the lower prevalence found in these social groups in other countries .*

**References**

1. Paul G Sanfilippo, Byoung-Sun Chu, Olivia Bigault, et al. Up to What Age is a Cyclopleged Refraction Required? Results from the Twins Eye Study Tasmania (TEST). Acta Ophthalmol. 2014; 92(6): e458-62.
2. Nisha S. Yeotikar, Ravi Chandra Bakaraju, P.S. Roopa Reddy, Kalyani Prasad. Cycloplegic refraction and non-cycloplegic refraction using contralateral fogging: a comparative study. Journal of Modern Optics. 2007;54(9):1317- 24.
3. French AN, Morgan IG, Burlutsky G, [Mitchell P](https://www.ncbi.nlm.nih.gov/pubmed/?term=Mitchell%20P%5BAuthor%5D&cauthor=true&cauthor_uid=23522969), [Rose KA](https://www.ncbi.nlm.nih.gov/pubmed/?term=Rose%20KA%5BAuthor%5D&cauthor=true&cauthor_uid=23522969). Prevalence and 5- to 6-year incidence and progression of myopia and hyperopia in Australian schoolchildren. Ophthalmol. 2013;120(7):1482-91.
4. French AN, O'Donoghue L, Morgan IG, [Saunders KJ](https://www.ncbi.nlm.nih.gov/pubmed/?term=Saunders%20KJ%5BAuthor%5D&cauthor=true&cauthor_uid=22562516), [Mitchell P](https://www.ncbi.nlm.nih.gov/pubmed/?term=Mitchell%20P%5BAuthor%5D&cauthor=true&cauthor_uid=22562516), [Rose KA](https://www.ncbi.nlm.nih.gov/pubmed/?term=Rose%20KA%5BAuthor%5D&cauthor=true&cauthor_uid=22562516). Comparison of refraction and ocular biometry in European Caucasian children living in Northern Ireland and Sydney, Australia. Invest Ophthalmol Vis Sci. 2012; 53(7):4021-31.
5. Elvis Ojaimi, Kathryn A Rose, Ian G Morgan, et al. Distribution of Ocular Biometric Parameters and Refraction in a Population-Based Study of Australian Children. Invest Ophthalmol Vis Sci. 2005;46(8):2748-54.
6. Junghans BM, Crewther SG. Prevalence of myopia among primary school children in eastern Sydney. Clin Exp Optom. 2003; 86(5):339-45.
7. Junghans BM, Crewther SG. Little evidence for an epidemic of myopia in Australian primary school children over the last 30 years. BMC Ophthalmol. 2005; 5(1): 1.
